# Allograft Deltoid Ligament Reconstruction and Z-Lengthening Fibular Osteotomy for Residual Valgus Instability After Ankle Fracture Fixation: A Case Report

**DOI:** 10.3390/healthcare14040522

**Published:** 2026-02-18

**Authors:** Sreenivasulu Metikala, Madana Mohana R. Vallem, Khalid Hasan

**Affiliations:** Department of Orthopedics, School of Medicine, Virginia Commonwealth University, Richmond, VA 23289, USA; madana.vallem@vcuhealth.org

**Keywords:** ankle instability, fibular osteotomy, deltoid ligament, allograft reconstruction, syndesmotic malreduction

## Abstract

Residual valgus instability following ankle fracture fixation presents a reconstructive challenge, especially when medial soft tissue compromise precludes early deltoid ligament repair. Restoring medial stability, together with fibular length and syndesmotic alignment, is crucial for re-establishing joint congruity and preventing progressive deformity or degenerative complications. In this single-patient case report, we describe a novel technique combining the use of an allograft deltoid ligament reconstruction with a Z-lengthening distal fibular osteotomy in a young adult male who developed residual valgus instability after the lateral-only fixation of a Weber C ankle fracture–dislocation. The Z-lengthening osteotomy enabled the controlled, fluoroscopy-guided restoration of fibular length and the correction of syndesmotic malreduction. Concurrently, medial stabilization was achieved with a suspensory-and-aperture fixation allograft construct, providing a tensionable anatomic reconstruction of the deltoid complex. This integrated approach restored the alignment of the medial clear space and syndesmosis, resulting in a pain-free, stable ankle mortise. At the three-year follow-up, the patient maintained a stable reduction with no radiographic signs of post-traumatic arthritis. The technique offers a reproducible, joint-preserving solution that merges mechanical correction with biological reconstruction to restore circumferential ankle stability and facilitate functional rehabilitation after complex ankle fracture fixation.

## 1. Introduction

Residual valgus instability after ankle fracture fixation is a challenging and often under-recognized complication. It is most frequently encountered in high-energy ankle fracture–dislocations involving a suprasyndesmotic lateral malleolar fracture (Weber C), disruption of the distal tibiofibular ligamentous complex, and a complete deltoid ligament rupture [1]. Successful management in the acute setting depends on the accurate restoration of fibular anatomy and syndesmotic alignment, along with the re-establishment of medial stability through primary deltoid repair. Failure to achieve these goals has substantial biomechanical consequences. As shown by Ramsey and Hamilton, even minimal lateral talar shift dramatically reduces the tibiotalar contact area and increases joint stress [2]. Clinical studies further reinforce this, with Ovaska and colleagues identifying fibular malposition as the leading cause of syndesmotic malreduction and a major reason for early reoperation after ankle fracture fixation [3]. CT-based reviews confirm that syndesmotic malreduction remains common and often underappreciated intraoperatively [4].

In severe injury patterns, especially when patient presentation is delayed, the medial soft tissues may undergo necrosis. This renders the skin unsafe for surgical incision and prevents primary deltoid repair. In such circumstances, lateral-only fixation may be the only viable initial strategy, but it often proves insufficient as a surrogate for medial stabilization. When the deltoid ligament is torn and left unrepaired, the talus may drift laterally under axial load, resulting in recurrent medial clear space widening and functional instability [5,6]. Recent biomechanical data also show that isolated deltoid insufficiency contributes to syndesmotic instability, further emphasizing its role in maintaining mortise congruity [7].

Delayed presentations introduce additional complexity. Chronic scarring, attenuation, or resorption of the deltoid ligament frequently renders it irreparable, necessitating alternative reconstructive options. Allograft-based anatomic deltoid reconstruction has been shown to effectively restore medial restraint and improve mortise congruity, particularly when combined with the correction of underlying osseous malalignment [5,8,9]. Recent systematic reviews further confirm favorable outcomes and the restoration of stability [10,11,12].

When fibular shortening or malrotation persists, a corrective osteotomy becomes essential to re-establish the normal geometry of the ankle mortise. The Z-lengthening distal fibular osteotomy provides a multiplanar platform capable of controlled correction of length, rotation, and translation. Barg and colleagues demonstrated that this osteotomy reliably restored fibular alignment in cases of malunion, with favorable clinical and radiographic outcomes [13]. Modern analyses further support corrective fibular osteotomy as an effective method for restoring ankle congruity and improving clinical outcomes [14,15]. Achieving precise lengthening can be technically demanding. The push–pull distraction technique described by Woodward et al. allows controlled millimetric length restoration while maintaining stable plate–bone contact [16].

This report describes a combined reconstructive approach for residual valgus ankle instability in the subacute postoperative setting of a Weber C fracture–dislocation. The individual components of this treatment—fibular lengthening osteotomy and deltoid ligament reconstruction—have been described previously. In the present case, however, both procedures were necessary and were performed together to resolve the persistent instability that followed the lateral-only fixation. The Z-lengthening distal fibular osteotomy restored fibular length and syndesmotic alignment. Meanwhile, the allograft deltoid ligament reconstruction addressed medial ligament insufficiency when primary repair was no longer feasible. By treating both osseous malalignment and ligamentous deficiency in a single revision procedure, ankle stability was restored. The preservation of ankle alignment and function at three-year follow-up suggests that this combined approach may provide lasting correction in selected cases.

## 2. Case Description

**Figure 1:**
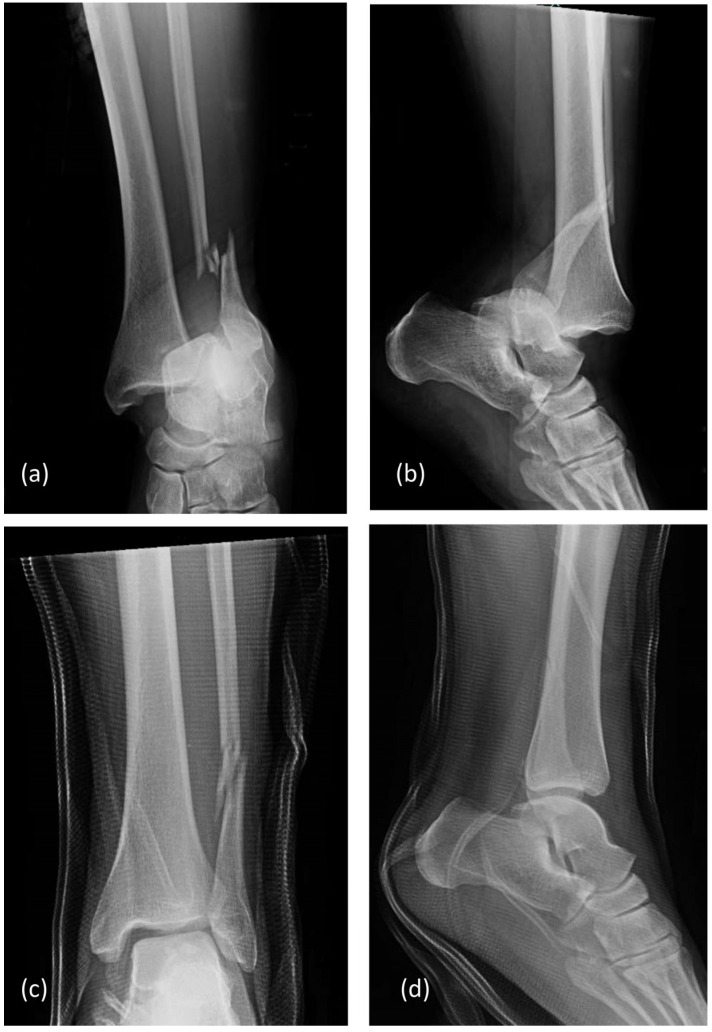
Injury radiographs of left ankle AP (**a**) and lateral (**b**) views showing posterolateral fracture dislocation. Post-reduction radiographs AP (**c**) and lateral (**d**) views showing restoration of gross alignment.

A 37-year-old male presented with left ankle pain and deformity following an altercation in a bar. He described experiencing unexpected extreme twisting forces with an immediate inability to bear weight. Initial emergency department evaluation showed swelling, deformity, and tenderness. Radiographs revealed a Weber C distal fibula fracture with posterolateral dislocation, including lateral talar shift, medial clear space widening, and tibiofibular diastasis. Closed reduction restored gross alignment, and the ankle was protected in a splint (Figure 1). The patient was then taken into police custody and did not return until four days later.

**Figure 2:**
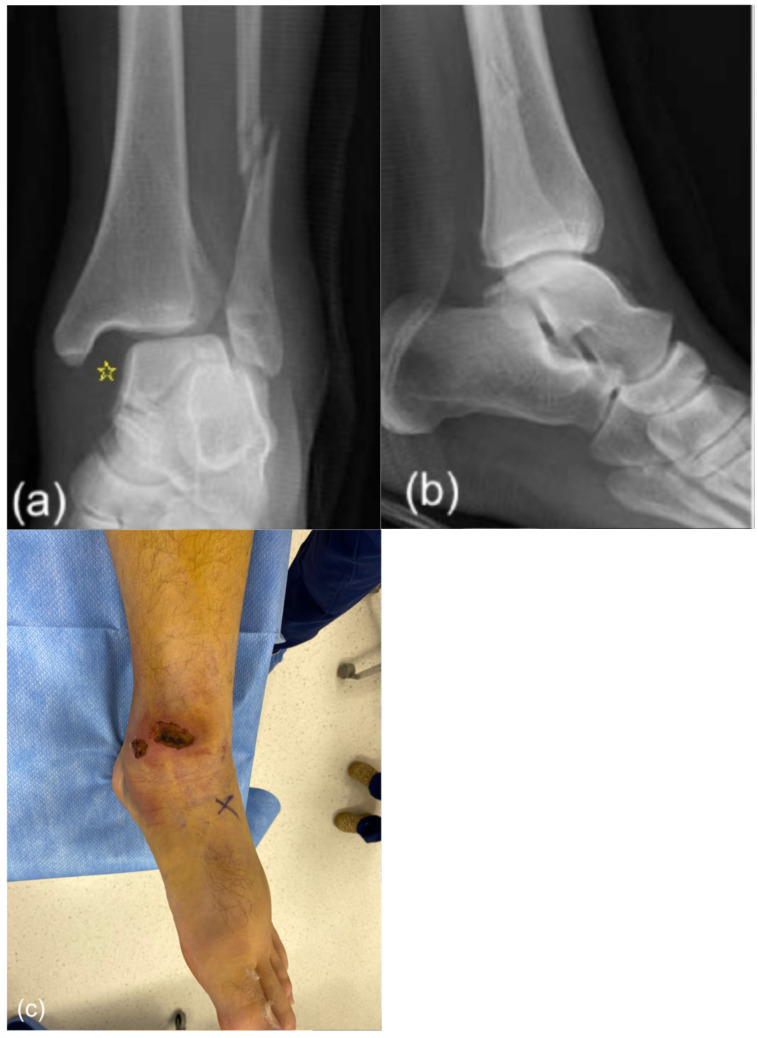
Preoperative AP (**a**) and lateral (**b**) radiographs on day 8 showing loss of tibio-talar alignment with widening of medial clear space (asterisk) and syndesmosis; (**c**) clinical picture showing full-thickness necrosis on the medial side.

At that visit, evolving medial skin necrosis overlying the deltoid region was noted. Repeat radiographs showed maintained tibio-talar alignment but persistent widening of the medial clear space and syndesmotic interval. Surgery was recommended, but because of custody-related delays, it was not performed until day eight. By that time, the patient had lost radiographic alignment despite splint protection, and the medial skin necrosis had progressed (Figure 2) to the point that a surgical incision could not be safely performed, rendering primary deltoid repair infeasible. A lateral-only fixation strategy was therefore selected.

**Figure 3:**
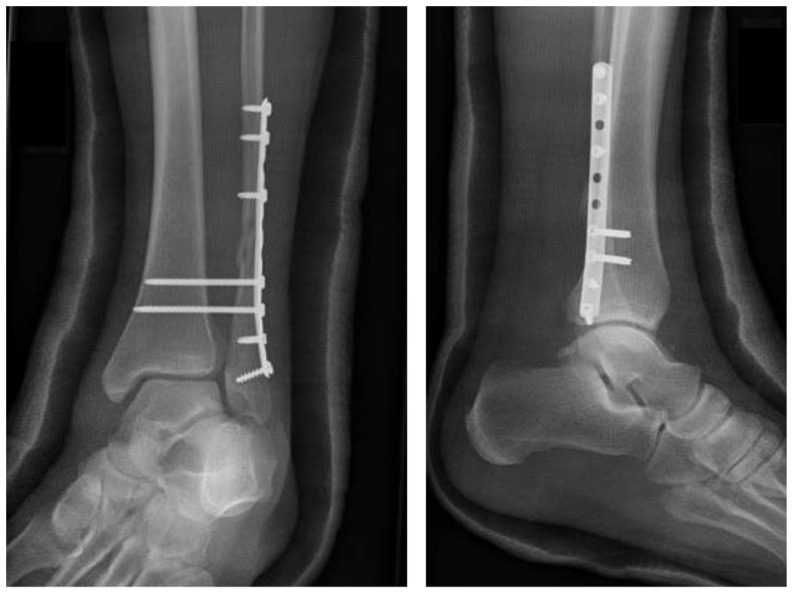
Postoperative radiographs at 6 weeks showing lateral-only fixation using 1/3rd tubular plate and syndesmosis screws with maintenance of alignment and early healing of distal fibular fracture.

Through a direct lateral approach, the distal fibula fracture was reduced and stabilized with a one-third tubular plate, and two syndesmotic screws were placed through the plate. Intraoperative fluoroscopic stress testing demonstrated a stable mortise in neutral and varus positions, but revealed mild valgus talar tilt under valgus stress that was attributed to the unrepaired deltoid ligament. A short-leg non-weight-bearing cast in slight varus was applied. At six weeks, the patient had no ankle pain and radiographs showed early fibular fracture healing (Figure 3).

**Figure 4:**
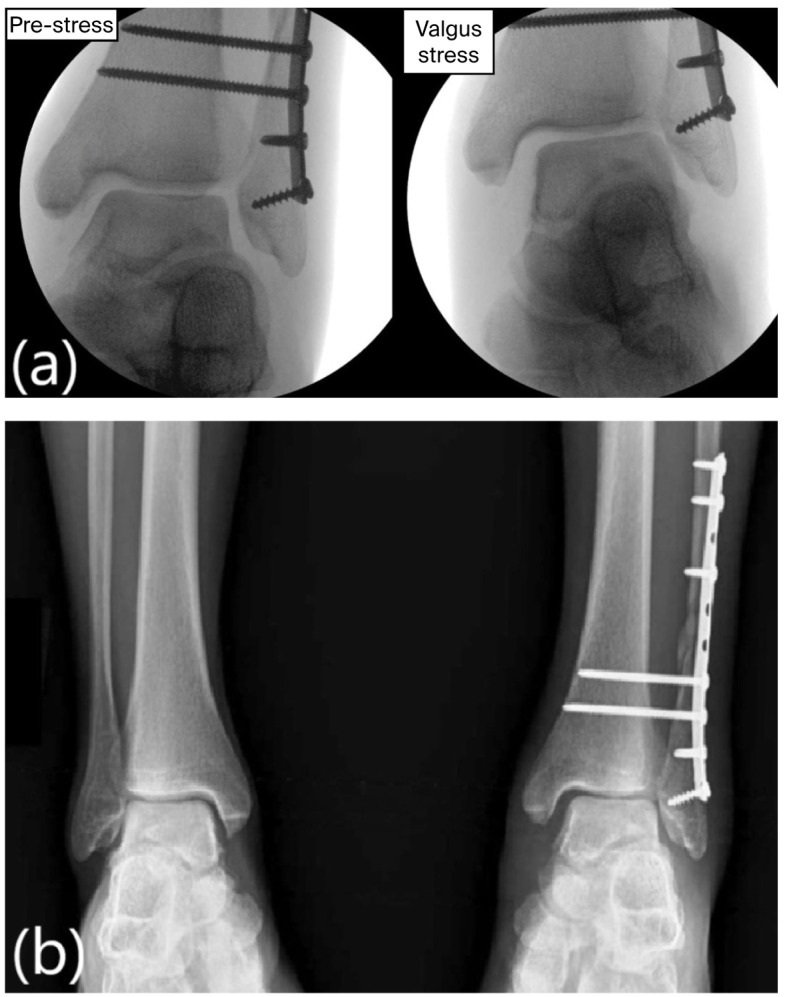
Stress fluoroscopy (**a**) in the office at week 8 postop showing persistent medial instability. Bilateral standing AP radiograph (**b**) showing subtle widening of medial clear space and fibular shortening.

Progressive weight bearing in a controlled-ankle-motion (CAM) boot was then initiated along with physical therapy. Within two weeks, however, the patient returned to the clinic with medial pain and a subjective give-way sensation during weight bearing. Stress fluoroscopy reproduced the valgus talar tilt observed during the primary operation, indicating persistent medial instability (Figure 4a). Weight-bearing ankle radiographs, including a bilateral anteroposterior (AP) view, demonstrated relative shortening of the distal fibula, a positive dime sign, and recurrent medial clear space widening measuring approximately 5 mm (Figure 4b).

**Figure 5:**
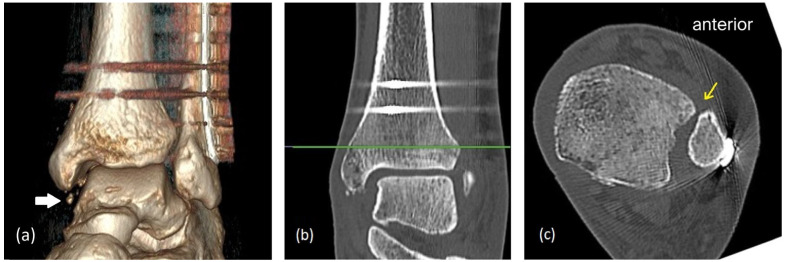
Postop CT scan showing avulsed fragments in the medial gutter (solid white arrow, (**a**)); anterior syndesmotic malreduction (yellow arrow, (**c**)), referenced at 1 cm proximal to the ankle joint line (green line, (**b**)).

A computed tomography (CT) scan confirmed syndesmotic malalignment with anterior positioning of the distal fibula within the incisura, mild fibular shortening, and ossified scar tissue occupying the medial gutter (Figure 5). These findings were consistent with fibular malreduction, deltoid ligament insufficiency, and recurrent syndesmotic instability. Given the patient’s ongoing symptoms and imaging abnormalities, revision surgery was recommended. 

## 3. Surgical Technique

Revision surgery was performed approximately three months after the index procedure under general anesthesia, with the patient positioned supine and a thigh tourniquet applied. By this time, the medial soft tissue envelope had completely healed without residual necrosis and was deemed suitable for surgical exposure. At the start of the procedure, fluoroscopic images of the contralateral ankle were obtained to serve as anatomic references for the restoration of fibular length and syndesmotic alignment during reconstruction.

The medial side was addressed first. An anteromedial incision was made along the course of the deltoid ligament, and an arthrotomy was performed while protecting the saphenous nerve. On entering the joint, dense scar tissue and bone debris were encountered filling the medial gutter. The deltoid ligament was completely deficient, with no viable fibers suitable for primary repair. Debridement was carried out until the talus was centered within the medial mortise. The planned bone tunnel sites were marked at the intercollicular groove of the medial malleolus, the anteromedial non-articular region of the talus, and the sustentaculum tali.

**Figure 6:**
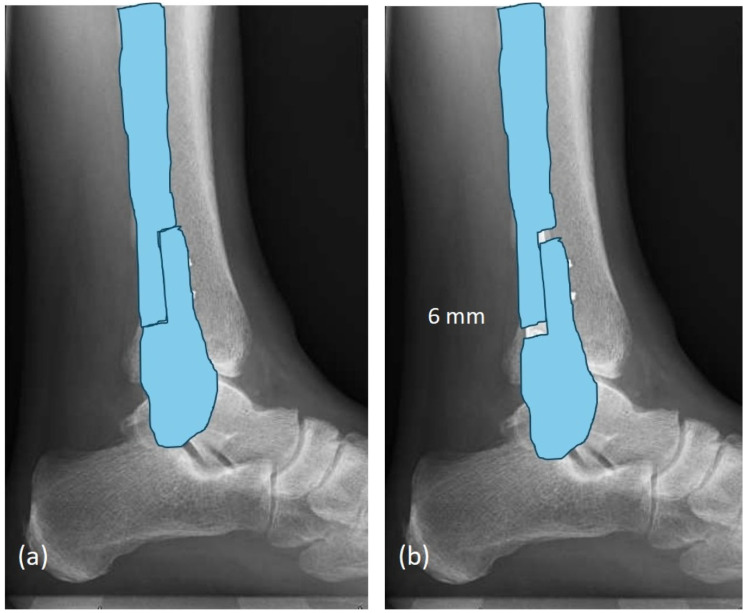
Preoperative plan of Z-lengthening osteotomy of distal fibula: (**a**) before and (**b**) after.

Attention was then redirected laterally. The previous lateral incision was reopened and extended distally with a gentle anterior curve. Subperiosteal dissection exposed the distal fibula, and the hardware from the index fixation was removed without difficulty. Fracture healing was noted, and no signs of infection were present. Scar tissue was cleared from the distal tibiofibular interval to allow visualization of the talar dome and incisura. A Z-shaped osteotomy, as per the preoperative plan (Figure 6), was created through the suprasyndesmotic portion of the distal fibula using multiple drill holes connected with a microsagittal saw under continuous irrigation.

**Figure 7:**
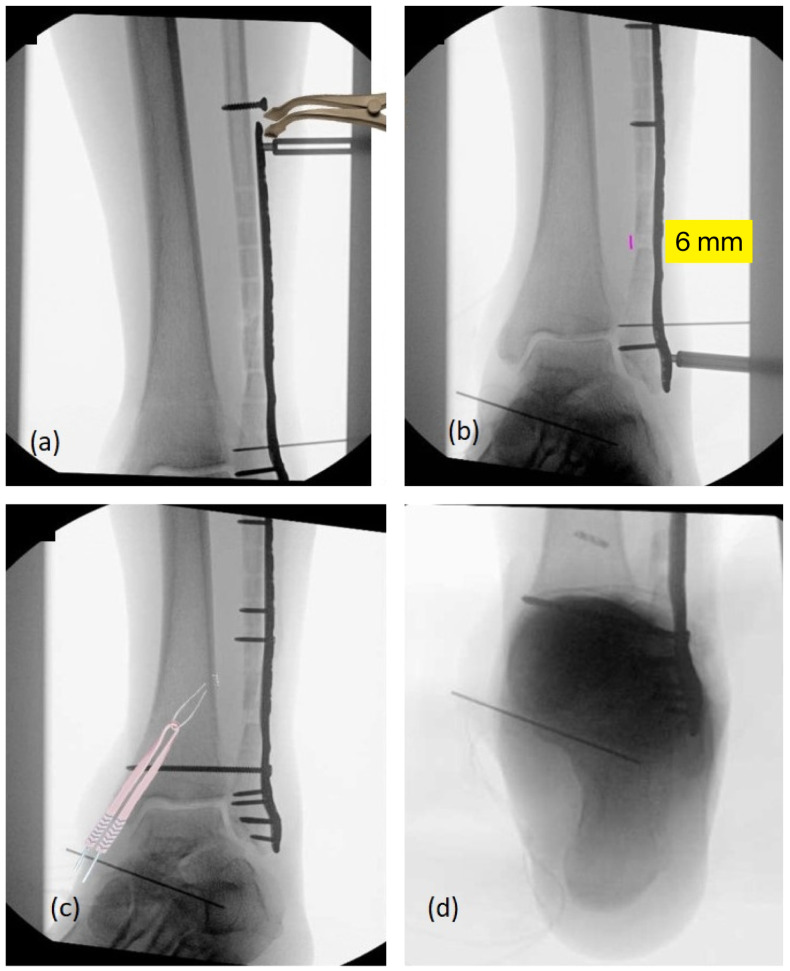
Intraoperative fluoroscopy images. (**a**) Push–pull screw with laminar spreader. (**b**) Six-millimeter length gain in distal fibula. (**c**) Allograft illustration into tibial tunnel with suspensory fixation. (**d**) Guide pin into sustentaculum tali for calcaneal bone tunnel.

A nine-hole variable-angle 2.7 mm locking plate (longer than the previous plate) was positioned along the lateral fibula and secured distally with locking screws. Proximal to the osteotomy, a 3.5 mm cortical screw was placed and used in conjunction with a laminar spreader positioned between the screw head and a threaded drill sleeve in the plate. This push–pull construct allowed for the controlled distraction of the osteotomy site. Under fluoroscopic guidance, gradual distraction was performed until approximately 6 mm of fibular length was restored, matching the anatomy of the contralateral side (Figure 7a,b). Once satisfactory length and alignment were achieved, the plate was secured proximally with multiple locking screws. Cancellous autograft harvested from a small oblique incision over the proximal lateral tibia was packed into the osteotomy site. The distal tibiofibular syndesmosis was then inspected directly. Interposed scar tissue within the incisura was debrided, and the syndesmosis was anatomically reduced under direct vision. Provisional fixation was obtained with two 1.6 mm Kirschner wires. After confirming the reduction fluoroscopically and through comparison with the contralateral ankle, a 3.5 mm cortical positional screw was placed across all four cortices. The provisional wires and the temporary push–pull screw were then removed.

The medial side was revisited to complete the deltoid reconstruction. A 5 × 150 mm semitendinosus allograft was prepared on the back table. Three bone sockets were drilled: a 30 × 5.5 mm socket in the medial malleolus at the intercollicular groove, and two 20 × 5 mm sockets in the talus and sustentaculum tali. The graft was looped through a suture button suspensory device and passed into the medial malleolar socket using a Beath pin. The button was delivered through a small anterior tibial incision and flipped securely against the cortex. The two free limbs of the graft were then docked into the talar and sustentacular sockets and secured with 4.75 mm biotenodesis screws (Figure 7c,d). Final graft tensioning was performed with the ankle in neutral and slight inversion by the sequential tightening of the suspensory sutures until medial stability was achieved.

All wounds were thoroughly irrigated and closed in layers. Local anesthetic was infiltrated around the incisions, and a well-padded posterior splint was applied. The patient was transferred to the recovery room in a stable condition with instructions to remain non-weight-bearing for six weeks.

## 4. Postoperative Care and Follow-Up

After surgery, the patient was placed in a well-padded posterior splint with the ankle maintained in neutral alignment and was instructed to remain non-weight-bearing for six weeks. Surgical sutures were removed after two weeks, and a short-leg cast was applied. At six weeks, radiographs demonstrated early healing of the Z-lengthening osteotomy, preserved fibular length, and stable syndesmotic alignment. The medial clear space remained symmetric to that of the contralateral ankle. The patient was transitioned to a CAM boot for protected weight bearing, and a structured physical therapy program was initiated. Early rehabilitation emphasized the restoration of ankle motion, followed by gradual strengthening and proprioceptive exercises. Weight bearing was advanced as tolerated, beginning with crutches and progressing to full weight bearing over the next month.

By four months, the patient was ambulating in regular footwear without assistive devices. He reported improvement in stability and complete resolution of his preoperative medial ankle pain. Clinical examination demonstrated a stable ankle mortise with no valgus tilt on stress testing. Radiographs confirmed healing of the osteotomy, restoration of fibular height, and the maintenance of syndesmotic reduction. He was cleared to resume full work duties and subsequently reported no episodes of instability or giving way.

**Figure 8:**
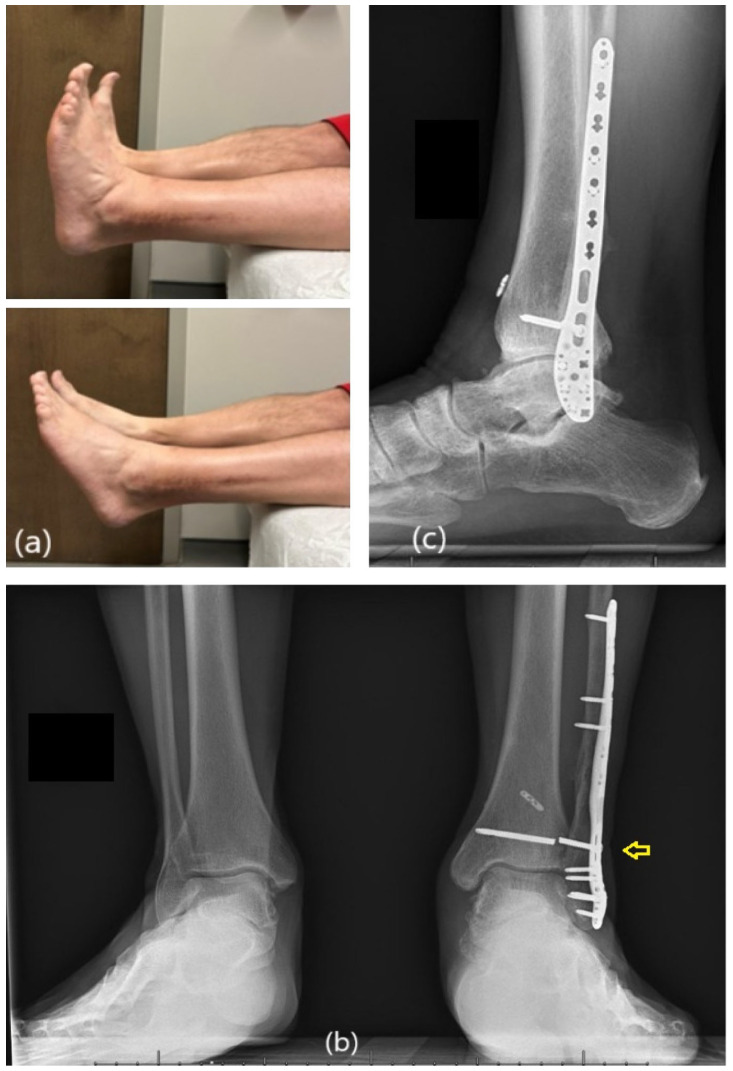
Final 3-year follow-up. (**a**) Clinical images showing functional ankle motion. Weight-bearing AP (**b**) and lateral (**c**) radiographs showing broken syndesmotic screw (yellow arrow) with congruent ankle mortise.

At the last three-year follow-up, he remained pain-free with full ankle range of motion and unrestricted daily activity. Radiographs showed a symmetric and stable mortise compared with the contralateral side, complete osteotomy healing, and no signs of post-traumatic osteoarthritis. The previously placed syndesmotic screw had fractured, as commonly observed, but overall alignment remained unchanged and clinically stable (Figure 8).

## 5. Discussion

Residual valgus ankle instability after fracture fixation is an uncommon but clinically significant complication that often results from a combination of medial ligament insufficiency and subtle fibular malreduction. In high-energy injuries, such as Weber C fracture–dislocations, damage to the deltoid ligament, the syndesmotic complex, and the distal fibula may coexist and contribute to ongoing instability [1]. In this case, medial skin necrosis at the time of the initial surgery prevented deltoid repair, leaving lateral fixation as the only option. Although intraoperative fluoroscopy indicated a stable mortise under neutral and varus stress, the ankle could not tolerate valgus loading once weight bearing was initiated. This led to recurrent talar tilt and symptoms of instability. This case demonstrates that, in the subacute revision setting, durable valgus stability can be achieved by addressing both osseous and ligamentous pathology in a single procedure. Specifically, the combination of the correction of fibular shortening and syndesmotic malreduction through a Z-lengthening osteotomy, along with anatomic deltoid ligament reconstruction, was necessary to restore ankle stability, as fixing either component alone would likely have been insufficient.

The deltoid ligament complex is the primary stabilizer of the medial ankle, resisting valgus angulation and external rotation of the talus. When torn and left unrepaired, the talus loses its medial buttress and becomes prone to lateral drift during physiologic loading. Persistent medial clear space widening is a well-recognized consequence of deltoid incompetence, and several authors emphasize the importance of restoring the deltoid ligament to prevent late instability and progressive deformity [5,6]. Modern studies reinforce the fact that isolated deltoid insufficiency can independently destabilize the syndesmosis, further compromising ankle stability [7]. In this case, medial soft tissue necrosis rendered the deltoid irreparable during the initial operation. Hence, it created a mechanical deficit that became clinically evident during rehabilitation.

Fibular integrity is equally essential. Ramsey and Hamilton biomechanically demonstrated that even 1 mm of lateral talar shift reduces the tibiotalar contact area by approximately 42%, dramatically increasing joint stresses and accelerating degenerative wear [2]. Subsequent 3D imaging studies confirm the sensitivity of the ankle mortise to subtle fibular malalignment and its effect on talar centering [17]. Although the initial fixation appeared acceptable on plain radiographs, subsequent imaging revealed subtle fibular shortening and anterior displacement of the distal fibula. These abnormalities disrupted the normal incisura–fibula relationship and contributed to persistent ankle instability.

Clinical studies support these observations. Ovaska et al. reported that syndesmotic malreduction accounted for the majority of early reoperations after ankle fracture fixation, with most malreductions resulting from fibular malposition—particularly shortening or anterior translation—rather than hardware failure [3]. CT-based studies also show high rates of occult malreduction despite standard techniques [4]. Medial ligament insufficiency exacerbates this problem by allowing lateral talar drift, thereby magnifying even minor errors in fibular alignment.

Given the high risk of unrecognized malreduction, obtaining intraoperative fluoroscopic comparison views of the contralateral ankle is crucial. Summers and colleagues described a reliable method of assessing syndesmotic reduction using comparison imaging from the uninjured ankle. They demonstrated that side-to-side fluoroscopic evaluation improves the accuracy of intraoperative assessment and helps prevent subtle rotational or translational errors [18]. In the present case, contralateral ankle images were used during revision surgery and played a key role in confirming appropriate fibular length and incisural alignment during reconstruction.

When fibular malreduction contributes to persistent instability, corrective osteotomy becomes necessary. The Z-lengthening osteotomy provides a multiplanar corrective platform that permits simultaneous restoration of fibular length, rotation, and translation. Barg et al. demonstrated that this technique reliably corrected fibular malunions and improved both radiographic alignment and clinical outcomes, with high union rates and low complication profiles [13], findings supported by more recent systematic analyses of fibular corrective osteotomies [14,15]. In the current case, the Z-lengthening osteotomy restored approximately 6 mm of fibular length and re-established the appropriate position of the distal fibula within the incisura.

Controlled lengthening through an osteotomy can be technically demanding. The push–pull construct described by Woodward et al., which uses a proximal cortical screw and a laminar spreader acting against a threaded drill sleeve, enables smooth, precise distraction while preserving plate-to-bone contact [16]. This technique was instrumental in achieving anatomic fibular restoration in the present case.

Medial instability was then addressed with allograft-based deltoid reconstruction, which is often necessary when delayed presentation or tissue necrosis preclude primary repair. Studies by Haddad and others have demonstrated that deltoid allograft reconstruction reliably restores medial restraint, centers the talus, and improves ankle stability in chronic or subacute settings [5,8]. In addition, systematic reviews support its effectiveness in restoring medial stability [10,11]. The use of a suspensory button construct at the medial malleolus, combined with aperture fixation in the talus and sustentaculum tali, permitted accurate anatomic re-creation of the deltoid ligament components and provided a tensionable, stable construct.

Fracture of the syndesmotic screw was observed at the final three-year follow-up. This is a common finding after the restoration of ankle motion and did not result in recurrent syndesmotic diastasis or clinical symptoms. Overall, the three-year follow-up highlights the importance of addressing both medial and lateral contributors to ankle stability. The patient demonstrated a symmetric ankle mortise with preserved fibular length without signs of degenerative change. This aligns with biomechanical observations by Michelson, who emphasized that restoring circumferential ankle stability reduces abnormal joint loading and may limit progression toward post-traumatic arthritis [19].

This case underscores several practical considerations in the management of persistent valgus instability after ankle fracture fixation. Medial soft tissue compromise should be recognized early, as delayed deltoid repair is often not feasible. Subtle fibular malreductions may not be apparent on routine radiographs and should prompt advanced imaging when instability persists [20]. Accurate intraoperative assessment, including contralateral comparison views, is crucial to prevent syndesmotic malalignment. In complex revision cases, the combined reconstruction of osseous and ligamentous stabilizers may be necessary.

This report has several limitations. It describes a single patient, which limits generalizability. Standardized patient-reported outcome measures were not collected, which limits objective comparisons with other studies. In addition, the described technique requires careful fluoroscopic assessment and carries a learning curve. The fixation strategies used here reflect current reconstructive practice but are not universally standardized. Their application may be influenced by allograft cost and availability. Despite these limitations, the case highlights the importance of evaluating both fibular alignment and medial ligament competence in patients with persistent valgus instability following ankle fracture fixation. Addressing osseous malalignment and ligament insufficiency simultaneously may help restore and maintain ankle stability and joint congruity when primary deltoid repair is no longer feasible.

## 6. Conclusions

Residual valgus instability may occur following operative fixation of high-energy ankle fracture–dislocations, especially when medial soft tissue compromise precludes primary deltoid ligament repair and when subtle fibular shortening and syndesmotic malreduction remain unrecognized. In this case, a combined approach using Z-lengthening distal fibular osteotomy and allograft deltoid ligament reconstruction restored ankle stability, as well as mortise congruity, with maintained correction and no degenerative changes at three-year follow-up. Although this report describes a single patient, these outcomes support the consideration of combined osseous and ligamentous reconstruction in appropriately selected patients who continue to demonstrate valgus instability after ankle fracture fixation. Further studies with larger cohorts are warranted to validate these findings.

## Data Availability

The data presented in this study are available on request from the corresponding author. The data are not publicly available due to privacy restrictions.
